# Antimicrobial prescriptions in cats in Switzerland before and after the introduction of an online antimicrobial stewardship tool

**DOI:** 10.1186/s12917-020-02447-8

**Published:** 2020-07-03

**Authors:** Alina Hubbuch, Kira Schmitt, Claudia Lehner, Sonja Hartnack, Simone Schuller, Gertraud Schüpbach-Regula, Meike Mevissen, Ruth Peter, Cedric Müntener, Hanspeter Naegeli, Barbara Willi

**Affiliations:** 1 Institute of Veterinary Pharmacology and Toxicology, Vetsuisse Faculty, University of Zurich, Winterthurerstrasse 260, CH-8057 Zurich, Switzerland; 2 Clinic for Small Animal Internal Medicine, Vetsuisse Faculty, University of Zurich, Winterthurerstrasse 260, CH-8057 Zurich, Switzerland; 3 Institute for Food Safety and Hygiene, Vetsuisse Faculty, University of Zurich, Winterthurerstrasse 272, CH-8057 Zurich, Switzerland; 4 Division of Small Animal Internal Medicine, Vetsuisse Faculty, University of Bern, Länggassstrasse 128, CH-3012 Bern, Switzerland; 5 Section of Epidemiology, Vetsuisse Faculty, University of Zurich, Winterthurerstrasse 270, CH-8057 Zurich, Switzerland; 6 Veterinary Public Health Institute (VPHI), Vetsuisse Faculty, University of Bern, Schwarzenburgstrasse 161, CH-3097 Liebefeld, Switzerland; 7 Division of Veterinary Pharmacology and Toxicology, Vetsuisse Faculty, University of Bern, Länggassstrasse 124, CH-3012 Bern, Switzerland

**Keywords:** Antimicrobial stewardship program, Prescription guidelines, Antibiotics, Prescription patterns, Companion animals, HPCIA, Highest priority critically important antimicrobial, One Health

## Abstract

**Background:**

Antimicrobial stewardship activities are essential to improve prudent antimicrobial use. The aim of the present study was to evaluate changes in antimicrobial prescriptions in cats after the introduction of prudent use guidelines promoted by an online antimicrobial stewardship tool (AntibioticScout.ch) in Switzerland. Data from 792 cats presented to two university hospitals and 14 private practices in 2018 were included and compared to 776 cases from 2016. Cats were diagnosed with acute upper respiratory tract disease (aURTD), feline lower urinary tract disease (FLUTD) and abscesses. Clinical history, diagnostic work-up and antimicrobial prescriptions (class, dosage, duration) were assessed. Type and proportions [95% confidence intervals] of antimicrobial prescriptions were compared between the two evaluation periods and a mixed effects logistic regression model was applied to evaluate compliance with Swiss prudent use guidelines.

**Results:**

From 2016 to 2018, the proportion of antimicrobial prescription in all included cases decreased from 75.0% [71.8–78.0] to 66.7% [63.3–69.9]; this decrease was most pronounced for treatments at university hospitals (67.1% [59.5–74.0] to 49.3% [40.9–57.8]) and for cats with FLUTD (60.1% [54.6–65.4] to 48.8% [43.2–54.4]). Use of 3rd generation cephalosporins in private practices declined from 30.7% [26.5–35.1] to 22.1% [18.4–26.2], while overall use of non-potentiated aminopenicillins increased from 19.6% [16.4–23.0] to 27.8% [24.1–31.9]. In cases where antimicrobial therapy was indicated, compliance with guidelines did not increase (33.3% [26.6–40.6] to 33.5% [27.2–40.2]), neither at universities nor in private practices. On the other hand, antimicrobial treatment was more often withheld in cases with no indication for antimicrobial therapy (35.6% [30.1–41.4] to 54.0% [47.6–60.4]); this was found for private practices (26.7% [20.8–33.4] to 46.0% [38.4–53.7]) and for aURTD cases (35.0% [26.5–44.2] to 55.4% [44.7–65.8]).

**Conclusions:**

Overall proportions of antimicrobial prescription, unjustified antimicrobial therapy and, in private practices, use of 3rd generation cephalosporins decreased from 2016 to 2018 for the investigated feline diseases. However, overall compliance with Swiss prudent use guidelines was still low, implying that further efforts are required to foster prudent antimicrobial use in cats.

## Background

A One Health approach is required to combat the development and spread of antimicrobial-resistant bacteria [1, 2]. Antimicrobial use is thought to be a major driving force towards antimicrobial resistance [3, 4] and over-prescription of antimicrobials seems to be common in human and veterinary medicine [5–9]. The largest proportion of antimicrobials sold in Europe are used to treat food producing animals and the role of companion animals in this context has been neglected for a long time [10]. However, highest priority critically important antimicrobials (HPCIAs) are commonly administered to companion animals [11–13]. In cats, the extensive use of 3rd generation cephalosporins, particularly the long-lasting cefovecin, is problematic [12–18]. This can foster the development and spread of antimicrobial resistant microorganisms in veterinary patients, e.g. extended-spectrum beta-lactamase producing *Enterobacteriaceae* [19, 20]. Numerous reports support a transmission of resistant bacteria from companion animals to humans, underlining the need to also promote prudent antimicrobial use in small animal medicine [21–27].

Antimicrobial stewardship programs aim to preserve the effectiveness of available antimicrobial agents and include different approaches, such as staff education, infection prevention and control, surveillance of antimicrobial resistance and health-care associated infections, propagation of prudent antimicrobial use and restrictions for HPCIAs [28, 29]. A common tool to enhance prudent antimicrobial use are prescription guidelines. In veterinary medicine, various countries and organizations have developed guidelines that are adapted to national requirements or local needs [30–35]. To date, only very few studies have investigated the impact of those guidelines on antimicrobial prescription in companion animals. A decrease of antimicrobial prescriptions after the introduction of prudent use guidelines has been reported in Flemish small animal practices and in a veterinary teaching hospital in Canada [9, 36]. In a Europe-wide survey, veterinarians in countries with national policies for antimicrobial use, as for example Sweden, seemed to prescribe critically important antibiotics less frequently than in countries without such policies [11]. In Denmark, 65% of companion animal practitioners reported in a questionnaire-based survey that the Danish national antibiotic use guidelines had influenced their prescription habits [37].

In Switzerland, the ban of antimicrobial growth promoters in 1999 together with other regulations and activities (e.g. drug recording requirements in food-producing animals, herd management by specialized veterinarians, disease eradication and vaccination programs) were associated with a reduction in antimicrobial use [38]. Since November 2015, a national Strategy on Antimicrobial Resistance (StAR) reinforces these measures [39]. Antimicrobial sale numbers in Switzerland showed a decrease of 41.1% between 2012 and 2018, while sales of antimicrobials exclusively registered for companion animals dropped by 13.4% [40]. However, population-adjusted antimicrobial sales for food-producing animals are still high compared to countries such as Sweden, Norway, Finland and Iceland, which emphasizes the need for further actions [10].

In December 2016, an online antimicrobial stewardship tool (AntibioticScout.ch) was introduced to promote Swiss guidelines on prudent antimicrobial use. This tool contains specific recommendations on antimicrobial prescription for various disease complexes [41–43]. AntibioticScout.ch has been published and disseminated through different channels, including the official journal of the Swiss Veterinary Society [42, 43], in newsletters of the Swiss Veterinary Society, on the website of the Federal Food Safety and Veterinary Office [44], on the website of the Institute of Veterinary Pharmacology and Toxicology of the University of Zurich [41], and in numerous continued education events for practitioners. The tool is accessed between 300 and 600 times a day (status March 2020) and introduced to each veterinary student in Switzerland. A previous study evaluated the a priori compliance with the guidelines for cats with acute upper respiratory tract disease (aURTD), feline lower urinary tract disease (FLUTD) and abscesses in Switzerland in 2016, before implementation of AntibioticScout.ch. The study reported an overall poor compliance of 17–24%, and 3rd generation cephalosporins were the second most commonly prescribed antibiotic class in these patients [45]. The present study is a follow-up investigation to examine changes of antimicrobial prescriptions in cats in Switzerland after launching the online antimicrobial stewardship tool AntibioticScout.ch. For this purpose, data for 2016 and 2018 from 14 private veterinary practices and two university hospitals on cats with aURTD, FLUTD and abscesses were compared and prescription patterns and compliance with guidelines evaluated.

## Results

### Case characteristics

A total of 792 cases (aURTD, *n* = 244; FLUTD, *n* = 324; abscesses, *n* = 224) was included in 2018 and compared to 776 cases from 2016 [45]. Case characteristics are given in Table 1. There was no difference in the proportion of cases presented to university hospitals compared to private practices between 2016 and 2018. Moreover, sex and breed distribution for the three disease complexes was not different between the two evaluation periods (Table 1), as well as age distribution in cases with FLUTD and abscesses. However, cats suffering from aURTD in 2018 were significantly older compared to cats in 2016 (Additional file 1).

**Table 1:** Characteristics of cases in 2016 and 2018 for cats with aURTD^a^, FLUTD^b^ and abscesses

Parameter		aURTD^**a**^		FLUTD^**b**^		Abscesses	
		2016	2018	2016	2018	2016	2018
**Total number of cases**		*n* = 227	n = 244	*n* = 333	n = 324	*n* = 216	n = 224
		% [CI]^c^	% [CI]^c^	% [CI]^c^	% [CI]^c^	% [CI]^c^	% [CI]^c^
Treatment location	University hospital	18.9 [14.1–24.7]	11.5 [7.8–16.2]	39.0 [33.8–44.5]	35.8 [30.6–41.3]	0.0^d^	0.0^d^
	Private practice	81.1 [75.3–85.9]	88.5 [83.8–92.2]	61.0 [55.5–66.2]	64.2 [58.7–69.4]	100.0^d^	100.0^d^
Sex	Female	43.6 [37.1–50.3]	39.8 [33.6–46.2]	42.0 [36.7–47.5]	40.7 [35.3–46.3]	29.6 [23.6–36.2]	31.3 [25.2–37.8]
	Male	53.3 [46.6–59.9]	55.3 [48.9–61.7]	57.4 [51.8–62.7]	57.1 [51.5–62.6]	68.5 [61.9–74.7]	65.6 [59.0–71.8]
	Unknown	3.1 [1.2–6.3]	4.9 [2.6–8.4]	0.6 [0.1–2.2]	2.2 [0.9–4.4]	1.9 [0.5–4.7]	3.1 [1.3–6.3]
Breed	Purebred	19.4 [14.5–25.1]	20.9 [16.0–26.5]	20.7 [16.5–25.5]	21.6 [17.2–26.5]	6.5 [3.6–10.6]	7.6 [4.5–11.9]
	Mixed breed	75.8 [69.7–81.2]	75.0 [69.1–80.3]	74.2 [69.1–78.8]	73.5 [68.3–78.2]	88.0 [82.9–92.0]	87.5 [82.4–91.5]
	Unknown	4.8 [2.4–8.5]	4.1 [2.0–7.4]	5.1 [3.0–8.0]	4.9 [2.8–7.9]	5.6 [2.9–9.5]	4.9 [2.5–8.6]
Pretreatment	Yes^e^	9.3 [5.8–13.8]	8.2 [5.1–12.4]	8.1 [5.4–11.6]	8.6 [5.8–12.2]	3.2 [1.3–6.6]	2.7 [1.0–5.7]
	Unknown	2.6 [1.0–5.7]	2.0 [0.7–4.7]	2.4 [1.0–4.7]	1.2 [0.3–3.1]	0.9 [0.1–3.3]	0.4 [0.0–2.5]
Hospitalization	Yes^e^	15.9 [11.4–21.3]	10.7 [7.1–15.2]	36.0 [30.9–41.4]	36.7 [31.5–42.2]	6.0 [3.2–10.1]	9.8 [6.3–14.5]
Indication for AMU^f^	Yes	28.2 [22.4–34.5]	32.0 [26.2–38.2]	17.1 [13.2–21.6]	17.0 [13.1–21.5]	30.1 [24.1–36.7]	36.6 [30.3–43.3]
	No	**52.9 [46.1–59.5]**	**37.7 [31.6–44.1]**	31.8 [26.9–37.1]	33.6 [28.5–39.1]	30.6 [24.5–37.2]	21.0 [15.8–26.9]
	Unknown	18.9 [14.1–24.7]	30.3 [24.6–36.5]	51.1 [45.5–56.5]	49.4 [43.8–55.0]	39.4 [32.8–46.2]	42.4 [35.9–49.2]
Diagnostic work-up^g^	Yes^e^	11.9 [8.0–16.8]	8.2 [5.1–12.4]	52.3 [46.7–57.7]	56.8 [51.2–62.3]	NA^h^	NA^h^
Local wound treatment	Yes^e^	NA^h^	NA^h^	NA^h^	NA^h^	72.2 [65.7–78.1]	81.3 [75.5–86.1]
	Unknown	NA^h^	NA^h^	NA^h^	NA^h^	5.6 [2.9–9.5]	5.8 [3.1–9.7]
Drainage	Yes^e^	NA^h^	NA^h^	NA^h^	NA^h^	15.3 [10.8–20.8]	21.4 [16.2–27.4]

Non-overlapping 95% confidence intervals are shown in bold; Data from cases from 2016 has been published previously [45]; ^a^aURTD, acute upper respiratory tract disease; ^b^FLUTD, feline lower urinary tract disease; ^c^CI, 95% confidence interval; ^d^Cases with abscesses were only recruited in private practices (see methods); ^e^Values for the category “no” (reference group) are not shown; ^f^AMU, antimicrobial use; ^g^PCR for feline herpesvirus-1 and feline calicivirus in cases with aURTD; sediment analysis or culture of aseptically collected urine in cases with FLUTD; ^h^NA, not applicable

The proportion of cases that had been pretreated with antibiotics or those hospitalized was not different between 2016 and 2018 for the three disease complexes (aURTD, FLUTD and abscesses, Table 1). As in 2016 [45], the proportion of cases pretreated with antibiotics or hospitalized was higher at the university hospitals compared to private practice cases in 2018 (university hospitals vs. private practices, proportion [95% confidence interval (CI)]: pretreatment: 20.1% [13.9–27.6] vs. 3.9% [2.5–5.6]; hospitalization: 67.4% [59.1–74.9] vs. 10.8% [8.5–13.5]). In the pretreated cases, the antimicrobial class was either continued (2016: 25.5% [14.7–39.0]; 2018: 42.6% [29.2–56.8]) or switched (2016: 30.9% [19.1–44.8]; 2018: 31.5% [19.5–45.6]) or antibiotic treatment was stopped (2016: 23.6% [13.2–37.0]; 2018: 22.2% [12.0–35.6]). In some cases the antimicrobial class used for pretreatment was unknown (2016: 20.0% [10.4–33.0]; 2018: 3.7% [0.5–12.7]).

### Diagnostic work-up

The proportion of cases with aURTD tested for feline herpesvirus-1 and feline calicivirus by PCR did not differ between 2016 and 2018 (Table 1). As in 2016 [45], this diagnostic work-up was more commonly performed at the university hospitals compared to private practices in 2018 (university hospitals: 57.1% [37.2–75.5]; private practices: 1.9% [0.5–4.7]).

Urine sediment analysis or bacterial culture from an aseptically collected urine were performed in similar proportions of cases with FLUTD in 2016 and 2018 (Table 1). The proportion of cases receiving this diagnostic work-up increased in private practices from 2016 to 2018 (27.1% [21.1–33.8] to 43.8% [36.9–50.8]). Despite this increase, diagnostic work-up in cats with FLUTD was still more common at university hospitals (80.2% [71.7–87.0]) compared to private practices in 2018. Among cases with diagnostic work-up, a total of 36.9% [29.4–45.0] were diagnosed with bacteriuria in 2018 and similar numbers were obtained in 2016 (37.0% [29.6–44.8]).

### Antimicrobial prescriptions in 2016 and 2018 overall

**Fig. 1:**
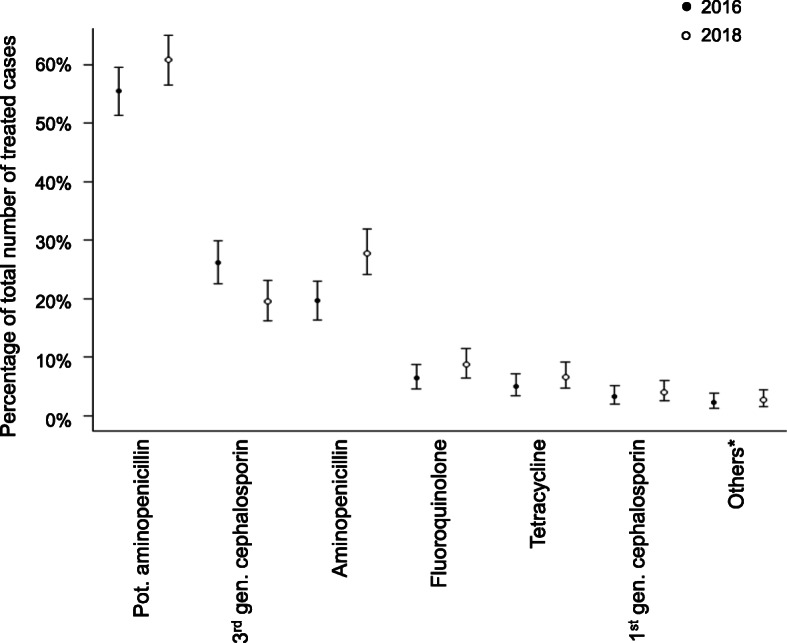
Comparison of antimicrobial classes prescribed in 2016 and 2018. Percentages of prescribed antimicrobial classes (dots) per total number of cases with antimicrobial treatment in 2016 (*n* = 582, filled dots) and 2018 (*n* = 528, empty dots) and corresponding 95% confidence intervals (lines). *Others (antimicrobial classes used in ≤ 2% of prescriptions) included amphenicoles, lincosamides, macrolides, penicillins, nitroimidazoles. Gen., generation; pot., potentiated

Proportions of prescribed antimicrobial classes in 2016 and 2018 are shown in Fig. 1. Details on antimicrobial prescriptions for all cases in 2016 and 2018, and data separated for university hospitals and private practices, are shown in Additional file 2.

Considering all cases of cats with aURTD, FLUTD or abscesses, the proportion of cases receiving antimicrobial treatment decreased from 2016 to 2018 (2016: 75.0% [71.8–78.0]; 2018: 66.7% [63.3–69.9]); this decline was distinct at the university hospitals (2016: 67.1% [59.5–74.0] of all cases; 2018: 49.3% [40.9–57.8] of all cases), but some reduction was also evident in private practice (2016: 77.3% [73.7–80.6] of all cases; 2018: 70.5% [66.8–74.0] of all cases). In 2018, the proportion of cases receiving antimicrobial treatment was higher in private practices compared to university hospitals.

When considering all cases receiving antimicrobials, the proportion of cases treated with HPCIAs did not change from 2016 to 2018 (2016: 33.0% [29.2–37.0]; 2018: 28.8% [25.0–32.9]), neither at the university hospitals (2016: 12.9% [7.4–20.4]; 2018: 12.7% [6.0–22.7]) nor in private practices (2016: 38.0% [33.6–42.6]; 2018: 31.3% [27.1–35.8]). On the other hand, the proportion of cases treated with 3rd generation cephalosporins decreased in private practices (2016: 30.7% [26.5–35.1] of treated cases; 2018: 22.1% [18.4–26.2] of treated cases), and overall, the proportion of cases treated with non-potentiated aminopenicillins increased (Fig. 1). Prescribed HPCIAs included 3rd generation cephalosporins, macrolides and quinolones; fourth or higher generation cephalosporins, ketolides, glycopeptides and polymyxins have not been prescribed to patients in this study. Combination or serial therapy was more common in 2018 than in 2016 (2016: 17.0% [14.0–20.3] of treated cases; 2018: 27.7% [23.9–31.7] of treated cases). Details on prescribed combination therapies are shown in Additional file 3.

### Antimicrobial prescriptions in 2016 and 2018 for cats with aURTD, FLUTD and abscesses

Details of antimicrobial prescriptions in cats with aURTD, FLUTD and abscesses are given in Table 2. From 2016 to 2018, the proportion of antimicrobial treatments in cats with aURTD did not change, but serial/combination therapy was more frequently used in 2018. Treatment duration in cats with aURTD was similar in 2016 compared to 2018 (Table 2).

**Table 2:** Antimicrobial prescriptions in 2016 and 2018 for cases with aURTD^a^, FLUTD^b^ and abscesses

Parameter		aURTD^**a**^		FLUTD^**b**^		Abscesses	
		2016	2018	2016	2018	2016	2018
**Total number of cases**		*n* = 227	*n* = 244	*n* = 333	*n* = 324	*n* = 216	*n* = 224
		% [CI]^c^	% [CI]^c^	% [CI]^c^	% [CI]^c^	% [CI]^c^	% [CI]^c^
Antimicrobial treatment	Yes^d^	77.1 [71.1–82.4]	67.6 [61.4–73.5]	**60.1 [54.6–65.4]**	**48.8 [43.2–54.4]**	95.8 [92.2–98.1]	91.5 [87.1–94.8]
**Details of antimicrobial treatment**		*n* = 175	*n* = 165	*n* = 200	*n* = 158	*n* = 207	*n* = 205
		% [CI]^c^	% [CI]^c^	% [CI]^c^	% [CI]^c^	% [CI]^c^	% [CI]^c^
Potentiated aminopenicillin		40.0 [32.7–47.7]	49.1 [41.2–57.0]	60.5 [53.4–67.3]	69.6 [61.8–76.7]	63.8 [56.8–70.3]	63.4 [56.4–70.0]
3rd generation cephalosporin		28.0 [21.5–35.3]	23.6 [17.4–30.9]	25.5 [19.6–32.1]	13.9 [8.9–20.3]	25.1 [19.4–31.6]	20.5 [15.2–26.7]
Aminopenicillin		23.4 [17.4–30.4]	29.1 [22.3–36.7]	11.5 [7.4–16.8]	20.9 [14.8–28.1]	24.2 [18.5–30.6]	32.2 [25.9–39.1]
Fluoroquinolone		4.0 [1.6–8.1]	8.5 [4.7–13.8]	12.5 [8.3–17.9]	15.8 [10.5–22.5]	2.4 [0.8–5.5]	3.4 [1.4–6.9]
Tetracycline		16.0 [10.9–22.3]	20.6 [14.7–27.6]	0.5 [0.0–2.8]	0.0 [0.0–2.3]	0.0 [0.0–1.8]	0.5 [0.0–2.7]
1st generation cephalosporin		0.6 [0.0–3.1]	0.0 [0.0–2.2]	3.0 [1.1–6.4]	1.3 [0.2–4.5]	5.8 [3.0–9.9]	9.3 [5.7–14.1]
Others^e^		4.0 [1.6–8.1]	3.6 [1.3–7.7]	0.5 [0.0–2.8]	0.0 [0.0–2.3]	2.4 [0.8–5.5]	3.9 [1.7–7.5]
HPCIAs^f^	Yes^d^	33.7 [26.8–41.2]	33.9 [26.8–41.7]	38.0 [31.2–45.1]	29.7 [22.7–37.5]	27.5 [21.6–34.2]	23.9 [18.2–30.3]
Serial/combination therapy	Yes^d^	**14.3 [9.5–20.4]**	**30.3 [23.4–37.9]**	13.0 [8.7–18.5]	19.6 [13.7–26.7]	23.2 [17.6–29.5]	31.7 [25.4–38.6]
**Treatment duration (days)**^**g**^		*n* = 140	*n* = 127	*n* = 178	*n* = 134	*n* = 153	*n* = 164
		Median (range)	Median (range)	Median (range)	Median (range)	Median (range)	Median (range)
		12 (4–37)	10 (1–21)	**13 (1–56)**^**h**^	**9.5 (1–42)**^**h**^	9 (1–28)	9 (2–28)

Non-overlapping 95% confidence intervals or comparisons with *p*-values < 0.05 (for treatment duration) are shown in bold; Data from cases from 2016 has been published previously [45]; ^a^aURTD, acute upper respiratory tract disease; ^b^FLUTD, feline lower urinary tract disease; ^c^CI, 95% confidence interval; ^d^Values for the category “no” (reference group) are not shown; ^e^Others (antimicrobial classes used in ≤ 2% of prescriptions) included amphenicoles, lincosamides, macrolides, penicillins, nitroimidazoles; ^f^HPCIAs, highest priority critically important antimicrobials; ^g^Treatment duration was unknown for numbers not listed (2016: *n* = 111; 2018: *n* = 103); ^h^*p*-value = 0.006

In 2018, less cases with FLUTD were treated with antimicrobials compared to 2016 and treatment duration decreased. Proportions of antimicrobial classes used in cats with FLUTD did not change (Table 2).

In cats with abscesses, the proportion of antimicrobial prescriptions was very high in 2018 (91.5%) and did not change compared to 2016. The antimicrobial classes prescribed were similar in both evaluation periods and treatment duration did not change between 2016 and 2018 (Table 2).

### Compliance with Swiss prudent use guidelines

To evaluate compliance with guidelines, cases were separated into those with and without indication for antimicrobial treatment. Cases with insufficient data for classification were excluded from analysis (private practices, 2016: *n* = 286, 2018: *n* = 304; universities, 2016: *n* = 12, 2018: *n* = 25). Exclusion of cats with FLUTD (2016: *n* = 170, 2018: *n* = 160) was mainly due to lack of diagnostic work-up, and in cats with aURTD (2016: *n* = 43, 2018: *n* = 74) and abscesses (2016: *n* = 85, 2018: *n* = 95) due to lack of documentation of clinical signs. Details on compliance with Swiss prudent use guidelines promoted by AntibioticScout.ch for the three disease complexes are shown in Table 3. Details for all cases and separated for the university hospitals and private practices are given in Additional file 4.

**Table 3:** Compliance with the guidelines in 2016 and 2018 for cases with aURTD^a^, FLUTD^b^ and abscesses

Category	aURTD^**a**^		FLUTD^**b**^		Abscesses	
	2016	2018	2016	2018	2016	2018
**AMU**^**c**^**indicated**	*n* = 64	*n* = 78	*n* = 57	*n* = 55	*n* = 65	*n* = 82
	% [CI]^d^	% [CI]^d^	% [CI]^d^	% [CI]^d^	% [CI]^d^	% [CI]^d^
Guidelines followed	10.9 [4.5–21.2]	16.7 [9.2–26.8]	38.6 [26.0–52.4]	45.5 [32.0–59.4]	50.8 [38.1–63.4]	41.5 [30.7–52.9]
Guidelines not followed						
a. Different dose/duration	6.3 [1.7–15.2]	2.6 [0.3–9.0]	1.8 [0.0–9.4]	5.5 [1.1–15.1]	24.6 [14.8–36.9]	34.1 [24.0–45.4]
b. Different antimicrobial class	75.0 [62.6–85.0]	67.9 [56.4–78.1]	54.4 [40.7–67.6]	38.2 [25.4–52.3]	21.5 [12.3–33.5]	22.0 [13.6–32.5]
c. Unjustified non-use	7.8 [2.6–17.3]	12.8 [6.3–22.3]	5.3 [1.1–14.6]	10.9 [4.1–22.2]	3.1 [0.4–10.7]	2.4 [0.3–8.5]
**AMU**^**c**^**not indicated**	*n* = 120	*n* = 92	*n* = 106	*n* = 109	*n* = 66	*n* = 47
	% [CI]^d^	% [CI]^d^	% [CI]^d^	% [CI]^d^	% [CI]^d^	% [CI]^d^
Guidelines followed	**35.0 [26.5–44.2]**	**55.4 [44.7–65.8]**	55.7 [45.7–65.3]	67.9 [58.3–76.5]	4.5 [0.9–12.7]	19.1 [9.1–33.3]
**Treatment duration**^**e**^	n = 140	n = 127	n = 178	n = 134	n = 153	n = 164
	% [CI]^d^	% [CI]^d^	% [CI]^d^	% [CI]^d^	% [CI]^d^	% [CI]^d^
Guidelines followed	89.3 [82.9–93.9]	88.2 [81.3–93.2]	44.4 [37.0–52.0]	54.5 [45.7–63.1]	39.2 [31.4–47.4]	39.6 [32.1–47.6]
Guidelines not followed						
a. Too long	10.7 [6.1–17.1]	10.2 [5.6–16.9]	50.6 [43.0–58.1]	38.8 [30.5–47.6]	57.5 [49.3–65.5]	56.1 [48.1–63.8]
b. Too short	0.0 [0.0–2.6]	1.6 [0.2–5.6]	5.1 [2.3–9.4]	6.7 [3.1–12.4]	3.3 [1.1–7.5]	4.3 [1.7–8.6]

Non-overlapping 95% confidence intervals are shown in bold; Data from cases from 2016 has been published previously [45]; ^a^aURTD, acute upper respiratory tract disease; ^b^FLUTD, feline lower urinary tract disease; ^c^AMU, antimicrobial use; ^d^CI, 95% confidence interval; ^e^Treatment duration is unknown for numbers not listed (2016: *n* = 111; 2018: *n* = 103)

Overall, the proportion of correct treatment decisions in cases in which antimicrobials were indicated did not increase from 2016 to 2018 (2016: 33.3% [26.6–40.6]; 2018: 33.5% [27.2–40.2]), neither at the university hospitals (2016: 26.8% [16.9–38.6]; 2018: 42.2% [27.7–57.8]) nor in private practices (2016: 37.4% [28.5–46.9]; 2018: 31.2% [24.3–38.7]). When cases without indication for antimicrobial treatment were analyzed, more cases were treated following Swiss guidelines in 2018 compared to 2016 (2016: 35.6% [30.1–41.4]; 2018: 54.0% [47.6–60.4]), i.e. the proportion of cases with unjustified antimicrobial therapies has declined. This increase in adherence to the guidelines in cases without indication for antimicrobial treatment was found in private practices (2016: 26.7% [20.8–33.4]; 2018: 46.0% [38.4–53.7]) and to some extend also at the university hospitals (2016: 55.6% [44.7–66.0]; 2018: 73.0% [61.4–82.6]).

When compliance with consensus guidelines was analyzed for cats with aURTD, a similar trend was seen. The proportion of correct treatment decisions in cases in which antimicrobials were indicated did not increase, but compliance increased in cases without an indication for antimicrobial treatment (Table 3). Compliance with treatment recommendations was generally low, mostly due to use of potentiated aminopenicillins instead of recommended non-potentiated aminopenicillins or doxycycline (Table 3). In cases with FLUTD or abscesses, the proportion of correct treatment decisions did not change between 2016 and 2018, neither in cases with or without indication for antimicrobial treatment (Table 3). Treatment duration and prescription of an antibiotic class not recommended by the guidelines, especially use of potentiated instead of non-potentiated aminopenicillins, was common in cats with FLUTD. In cats with abscesses, unnecessary treatments and treatments exceeding the recommended duration were frequent (Table 3).

### Mixed effects logistic regression model

A mixed effects logistic regression model was calculated for cases with and without an indication for antibiotic treatment (Table 3) to assess compliance of prescriptions with AntibioticScout.ch guidelines. In cases with an indication for antimicrobial treatment, the proportion of cases in which the guidelines were followed did not change from 2016 to 2018 (OR 1.0 [0.6–1.6]). In these cases, cats with FLUTD or abscesses were more often treated according to the guidelines than cats with aURTD, independently of the evaluation period (FLUTD: OR 5.1 [2.6–10.0]; abscess: OR 5.4 [2.9–10.0]). In cases without indication for antimicrobial treatment, guidelines were followed more frequently in the respective cases in 2018 than in 2016 (OR 2.4 [1.6–3.5]). In cases without indication for antimicrobial treatment, guidelines were followed less often in cats with abscesses compared to cats with aURTD (OR 0.1 [0.0–0.2]). On the other hand, in cats with FLUTD, a tendency towards more treatments in agreement with the guidelines was observed in comparison to cats with aURTD (OR 1.6 [1.0–2.7]). No significant interaction was found between year and disease complex.

## Discussion

The results of this study indicate an overall trend towards a more prudent antimicrobial prescribing practice in cats in Switzerland after the introduction of an online antimicrobial stewardship tool (AntibioticScout.ch) in December 2016. Between 2016 and 2018, overall proportions of antimicrobial use in cats with aURTD, FLUTD and abscesses declined, as well as prescriptions of 3rd generation cephalosporins in private practices. Furthermore, antimicrobials were more commonly withheld in cases where antimicrobial treatment was not recommended by the guidelines. This decrease of antimicrobial use is also supported by total sale statistics of antimicrobials registered for companion animals in Switzerland, which showed a decline of 4.9% during the same period [40]. Other studies also reported a reduction of antimicrobial prescriptions in both human and small animal medicine after the implementation of antimicrobial stewardship programs, underlining that these activities could have an impact on treatment strategies [9, 36, 46–49].

The reduction in use of 3rd generation cephalosporins from 30.7 to 22.1% in the private practices in 2018 is encouraging. The overall proportion of treatments with HPCIAs, however, did not decrease, which is possibly due to a slight increase in use of fluoroquinolones in 2018. Furthermore, the proportion of cases receiving HPCIAs was still very high, especially in private practices (31.3%). HPCIAs are not recommended for any of the investigated diseases and should only be used based on antimicrobial susceptibility testing. A significant decrease in use of 3rd generation cephalosporins in the university hospitals was not found (7.8 to 2.8%). However, this antibiotic class was already infrequently used in 2016.

Despite an overall decline in antimicrobial prescriptions, over-prescription was still common in 2018, especially in cats with abscesses. A total of 91.5% of the cats with abscesses in 2018 received antimicrobial therapy, compared to 67.6% of cases with aURTD and 48.8% of cats with FLUTD. This is striking because various studies from human medicine indicate that cure rates in uncomplicated skin abscesses are not influenced by antibiotic use as long as appropriate local wound therapy is applied [50–52]. Interestingly, 81.3% of the cats with abscesses in 2018 received a local wound treatment and 21.4% a drainage. This highlights that antimicrobials, although not indicated, are often prescribed in addition to local wound therapy in cats with abscesses. Fear of complications or owner dissatisfaction were listed as reasons for prescribing antimicrobials when not indicated in a recent interview-based study [16]. Veterinarians reported commonly perceiving pressure from owners to prescribe antimicrobials, especially in aggressive animals where diagnostic work-up and futher treatment options were limited by the patients’ behavior [16].

Potentiated aminopenicillins were still by far the most commonly applied antimicrobials in 2018. Use of potentiated aminopenicillins instead of non-potentiated aminopenicillins was also a common reason for lack of compliance with guidelines in cats with aURTD and FLUTD. It should be mentioned that, in 2018, no oral preparations of non-potentiated aminopenicillins registered for cats were on the market in Switzerland, whereas in 2016, a preparation was available on the market. This likely dissuaded veterinarians from following the guidelines in cases of uncomplicated urinary tract infections. In cases with aURTD, doxycycline is also listed as a first-line treatment. However, doxycycline was rarely prescribed despite its activity against *Chlamydia* sp. infection, possibly due to the potential risk of esophageal strictures in cats [53, 54].

Nevertheless, use of non-potentiated aminopenicillins recommended as first-line treatment option for all three disease complexes increased from 2016 to 2018 [41]. A comparably high percentage of non-potentiated aminopenicillins was prescribed in private practices in 2018. Preparations authorized for subcutaneous application accounted for most of these treatments. After discharge, veterinarians had to switch to oral preparations of potentiated aminopenicillins. Because this was classified as serial therapy in the study, the increase in serial/combination therapy in 2018, especially evident in private practices and in cases with aURTD, should therefore be interpreted with caution.

In cases with an indication for antimicrobial treatment, the proportion of correct treatment decisions did not increase. As described before, unjustified use of potentiated instead of non-potentiated aminopenicillins was a common reason for non-compliance. Difficulties to orally administer medications to cats and fear of non-compliance of the owner could be other reasons, favoring the use of 3rd generation cephalosporins, which can be injected as depot preparation (Convenia®, Zoetis, Delémont, CH) [55]. A recent interview-based study in Dutch companion animal veterinarians found that in case of aggressive animals, veterinarians prefered injectable antimicrobials and long-acting formulations [16]. According to this study, other reasons to select antimicrobials different from those recommended in the guidelines were personal preferences, a lack of familiarity with the guidelines or statements that the guidelines were unclear or impractical.

Treatment duration in our study was also often in disagreement with the guidelines, especially in cases with abscesses. Antibiotics were often prescribed for a longer duration than recommended. Overall, there is a lack of controlled studies to investigate optimal antimicrobial treatment duration in companion animal medicine, and recommendations in current guidelines are commonly extrapolated from human medicine [56]. This could result in believing that the recommended durations are not sufficient for a treatment success. Furthermore, shorter treatments may require check-up consultations to assess the success of treatment, thus causing additional costs to animal owners [17].

In cases where antimicrobials were not indicated, the proportion of treatments in compliance with guidelines increased from 35.6 to 54.0%, showing that unjustified antimicrobial use has declined for the investigated diseases. A study analyzing antimicrobial treatments of cats presented to Flemish small animal practices also noted a decrease of unjustified antimicrobial use, as well as an increase in compliance with the guidelines after their introduction [9]. In a study in human medicine, it was shown that the time period under investigation after implementation of guidelines has a great impact on the result: whereas 100% of the urinary tract infections were treated as recommended right after the introduction of guidelines, only 39% of prescriptions followed the guidelines after 1 year [57]. In our study, prescriptions were assessed in 2016 and 2018 over a 1 year period, which should provide a representative figure of the prescription habits. In the Flemish study, the follow-up period was not clearly specified [9].

Various differences in antimicrobial prescription habits between the university hospitals and private practices were apparent. First, as in 2016, HPCIAs were less commonly used at the university hospitals than in private practices [45]. Second, the proportion of cases treated with antimicrobials was lower at the university hospitals compared to private practices and the decrease in antimicrobial prescriptions was more pronounced. Third, antimicrobials were more commonly withheld at the universities in cases where antimicrobial treatment was not indicated. Finally, diagnostic work-up was more commonly performed at the universities as already reported in 2016 [45]. These findings indicate a trend towards a more restrictive use of antimicrobials at the university hospitals, which is important, as they could serve as role models for veterinary students and referring veterinarians. Despite this, overall compliance with the guidelines did not improve at the university hospitals from 2016 to 2018.

In private practices, urine sediment analysis or bacterial culture from an aseptically collected urine was more commonly performed in 2018 and could have contributed to the reduction of antimicrobial prescriptions in cases with FLUTD. Bacterial culture of urine should be further promoted, since it could prevent unnecessary antibiotic administrations. Costs of antimicrobial sensitivity testing and difficulties to obtain samples for bacterial cultures were reported to be reasons why veterinarians refrain from performing diagnostic work-ups [55, 58]. Bacterial cystitis is considered to be rare in cats, with reported prevalences of 3–15% in cats presented with FLUTD [56, 59–61]. Bacterial cystitis was much more common in cats with FLUTD in this study. This was also the case when diagnoses based on urine sediment analysis were excluded and only cases with a positive bacterial culture result were considered (31.0% in 2016, 33.3% in 2018). Our results are, however, in agreement with a study in Norway that also reported bacteriuria in 33% of cats presented with FLUTD [62].

In private practice, PCR testing for feline herpesvirus-1 and feline calicivirus in cases with aURTD was rarely performed in both evaluation periods (2018: 1.9% of cases; 2016: 1.1% of cases) [45]. The reason for this is unknown, but additional costs could be a limitation. One could argue that positive PCR test results for feline calicivirus do not affect treatment of cats with aURTD. However, in a confirmed feline herpesvirus-1 infection, topical or systemic antiviral treatment could be applied and potentially reduces the need for antibiotics [63].

Changes in prescription habits observed in this study are overall encouraging, although mis- and overuse of antimicrobials were still commonly observed. Further efforts are necessary to foster prudent antimicrobial use in companion animals in Switzerland. Since October 2019, antimicrobial prescriptions in companion animals have to be centrally registered in a national database (Informationssystem Antibiotika, IS ABV) [64]. This allows monitoring of trends in antimicrobial use on practice/clinic level and hopefully helps to increase awareness on prudent use in veterinary medicine in Switzerland. Information campaigns for animal owners in Switzerland on the importance of responsible antimicrobial use have been launched in 2015 [44], but they need to be futher developed to reach their goals. Better availability of first-line products on the Swiss market, and the development of easy to administer first-line antimicrobials and bedside diagnostics for antimicrobial sensitivity testings are future directions. Continued education programs for practitioners support these efforts and encourage experienced veterinarians to adapt their prescription habits to current guidelines.

This study has some limitations. The true impact of the online antimicrobial stewardship tool on antimicrobial prescribing of Swiss veterinarians cannot be unequivocally assessed, as different actions were implemented in Switzerland as part of the national StAR program to combat antimicrobial resistance starting in November 2015. These actions, together with a generally increased awareness of the importance of prudent antimicrobial use among veterinarians could have contributed to the prescription changes observed in this study. However, AntibioticScout.ch provides a user friendly decision support tool informing veterinary practitioners in a most direct and effective way. The investigated practices only represent a small proportion of practices in Switzerland and their participation was on a voluntary basis, which could have favored the inclusion of practices with more interest in prudent antimicrobial use. Limited information in the patient records especially in private practices impeded the evaluation of compliance with the guidelines. Almost 50% of the cases obtained from private practices were not suitable for assessment, mainly because of a lack of diagnostic work-up in cats with FLUTD or because the clinical symptoms in cats with aURTD and abscesses were not recorded. This could have caused a selection bias towards the inclusion of better documented cases and thus probably more severe or complicated cases, in which the adherence to prescription guidelines could have been hindered by comorbidities or other factors. Finally, data were analyzed by different evaluators in 2016 and 2018; because judgement of prudent antimicrobial use leaves some margin of interpretation, this could have had an influence on the results of the study.

## Conclusions

A trend towards a more prudent antimicrobial use in cats with aURTD, FLUTD and abscesses was found in Switzerland in 2018, after the implementation of the online antimicrobial stewardship tool AntibioticScout.ch. Overall antimicrobial use, prescription of 3rd generation cephalosporins in private practices and unjustified antimicrobial use decreased from 2016 to 2018 for the investigated disease complexes. Nevertheless, over-prescription of antimicrobials and use of HPCIAs was still common and overall compliance with the guidelines still poor. Antimicrobial stewardship activities should therefore be further promoted, and the availability of first-line antimicrobials with a convenient application in cats should be advanced.

## Methods

This follow-up study evaluated diagnostic work-up and antimicrobial prescription patterns for three feline disease complexes (aURTD, FLUTD, abscesses) in patients presented between January 1st and December 31st 2018 using identical methods and data extraction procedures from the same 14 veterinary practices and two university hospitals as in a previously published study [45]. AntibioticScout.ch was presented to the veterinarians working in the participating university hospitals and private practices at the beginning of the study in 2017. Only private practices using OblonData® (Amacker&Partner Informatik AG, Zurich, Switzerland) or Diana SUISSE® (Diana Software AG, Zurich, Switzerland) programs were eligible to participate. The electronic patient records of all animals treated during the two study periods (2016 and 2018) were extracted from the practice management system. At both university hospitals, reviewers had direct access to the electronical medical record system. A full text search was conducted for the predefined search terms (Table 4) and matches were manually reviewed. Cases were selected based on defined inclusion and exclusion criteria listed in Table 4 [45]. According to sample size calculation prior to the project, 92 patients per indication are required to detect a reduction in usage of an antimicrobial by 20%, given that this antimicrobial is used in 50% of the patients with a particular indication (calculated with the software WinEpiscope 2.0 [65], a power of 80 and 95% confidence). We therefore aimed to follow up on at least 100 patients per indication, preferably 200 patients, to adjust for clustering of observations in different practices or clinics. At both university hospitals, all cases that matched the criteria were included, whereas in private practices, 16 cases per indication and practice were chosen using the randomizer function of Microsoft® Excel (Microsoft Corporation, Washington, USA) to avoid overrepresentation of large practices. Only in private practices, cats with abscesses were included because they are rarely presented to university hospitals [45]. In two practices, only 10 and 14 cases with aURTD and 10 and 6 cases with FLUTD could be included in 2018 because of an insufficient number of patients matching the inclusion criteria. Data on medical history, clinical symptoms, diagnostic work-up and details on antimicrobial therapy and diagnosis were collected [45]. HPCIAs were defined according to the World Health Organization (WHO) to include third or higher generation cephalosporins, quinolones, macrolides, ketolides, glycopeptides and polymyxins [66]. Combination therapy was defined as prescription of two or more antimicrobial classes at a time, whereas serial therapy was defined as consecutive prescription of two or more antimicrobial classes [45]. Pretreated cases included both, cases referred from other institutions and treated for the actual disease, as well as cats presented to the same institutions and pretreated for unrelated diseases.

**Table 4:** Inclusion and exclusion criteria and search terms for aURTD^a^, FLUTD^b^ and abscesses

Indication	Inclusion criteria	Exclusion criteria	Search terms
**aURTD**ª	Nasal discharge with infectious or unknown etiology lasting no longer than two weeks	Evidence of fungal infection, neoplasia or involvement of the lower respiratory tract	Upper respiratory tract infection, rhinotracheitis, rhinitis, sinusitis, nasal discharge, sneezing, coughing, stridor, dyspnea, tachypnea, cat flu, herpes, calici, mycoplasma, chlamydia, laryngitis
**FLUTD**^b^	Stranguria, pollakiuria, periuria, pigmenturia or dysuria and a diagnosis of bacterial cystitis, bladderstones, urethrastones, urethral plugs, idiopathic cystitis or cystitis of unknown origin	Involvement of the upper urinary tract	Lower urinary tract disease, FLUTD^b^, pollakiuria, polyuria, anuria, stranguria, dysuria, hematuria, bloody urine, urinary stones, bladder stones, urolithiasis, concrements, cystitis, urethra obstruction, urinary tract infection, UTI, urinary incontinence
**Abscess**	Bite abscesses or abscesses of unknown origin	Anal gland abscesses, tooth root abscesses, foreign body abscesses	Abscess, bite wound, bite, pus

As published by Schmitt et al., 2019 [45]; ªaURTD, acute upper respiratory tract disease; ^b^FLUTD, feline lower urinary tract disease

Compliance with Swiss guidelines published in December 2016 in the online tool AntibioticScout.ch was evaluated [41–43]. For this purpose, indication, antimicrobial class, dose and treatment duration were evaluated and compared with the recommendations given in the guidelines as detailed in Table 5. Cases with insufficient documentation or diagnostic work-up to judge compliance with the guidelines were excluded from this analysis. Cases from 2016 and 2018 were reviewed by different evaluators. To assure consistency in judgment, the evaluators adhered strictly to the criteria listed in Table 5. Criteria not mentioned in the guidelines (e.g. the character of nasal discharge in cats with aURTD) were not considered. Undefined cases were discussed between evaluators to reach a consensus. To assess interrater reliability, 60 cases (30 cases from 2016 and 2018 each) were assessed independently by two evaluators with an observed Cohen’s kappa of 0.975 (standard error of 0.025; 95% CI of 0.926–1.000) [67].

**Table 5:** Swiss prudent use guidelines for cats with aURTD^a^, FLUTD^b^ and abscesses

Indication	Comment	Antibiotic	Dosage (mg/kg)	Application frequency	Treatment duration (days)
**aURTD**^**a**^	AMU^c^ is only indicated if poor general condition, fever (*≥ 39.5°C*), lethargy and/ or anorexia are present.	Doxycycline	10 / 5	SID^d^/BID^e^	5–14
		Amoxicillin	15–20	BID^e^/TID^f^	5–14
**FLUTD**^**b**^	AMU^c^ is only indicated in case of UTI^g^ (*defined as presence of bacteriuria*^*h*^*in patients with clinical signs of cystitis*^*i*^); AMU^c^ is not indicated in patients with indwelling urinary catheters; Complicated UTI^g^ is defined as infection caused by anatomical or functional changes or disorders of the immune system	**Uncomplicated UTI**^**g**^**:** Amoxicillin	11–15	BID^e^/TID^f^	5–7
		**Complicated UTI**^**g**^**:** Amoxicillin/ Clavulanic acid	12.5–20	BID^e^/TID^f^	5–28
**Abscess**	AMU^c^ is only indicated if signs of generalization (i.e. *fever ≥ 39.5°C*), poor general condition, severely contaminated wounds, and/or proximity to delicate tissues (e.g. joints, *thorax, abdomen, abscesses extending into deep tissue*) are present	Amoxicillin	15–20	BID^e^	5–7
		Amoxicillin/ Clavulanic acid	12.5–20	BID^e^	5–7
		Cefalexin	20–30	BID^e^/TID^f^	5–7
		Clindamycin	10–15	BID^e^	5–7
		Cefazolin	20	BID^e^	5–7

Consistent with the Swiss prudent use guidelines presented in December 2016 as previously published [45], additional details used to faciliate judgement are given in italics; in April 2019 the Swiss prudent use guidelines have been revised; ^a^aURTD, acute upper respiratory tract disease; ^b^FLUTD, feline lower urinary tract diseases; ^c^AMU, antimicrobial use; ^d^SID, once daily; ^e^BID, twice daily; ^f^TID, three times daily; ^g^UTI, urinary tract infection; ^h^Presence of bacteria in the urine sediment analysis or positive bacterial culture results from an aseptically collected urine sample (by cystocentesis or catheterization); ^i^Presence of stranguria, pollakiuria, periuria, pigmenturia or dysuria

For a comparison of the two evaluation periods (2016 and 2018), data were grouped into cases where antimicrobials were indicated (guidelines followed−/not followed) and cases where antimicrobials were not indicated (guidelines followed−/not followed, Table 6). This allowed to account for different proportions of cases presented with and without an indication for antimicrobial therapy in the two evaluation periods. Treatment decisions were defined as correct or incorrect based on Swiss guidelines.

**Table 6:** Details on categorization to evaluate compliance with Swiss prudent use guidelines

Category^**a**^	Explanation
**AMU**^**b**^**indicated**	
Guidelines followed	Antimicrobial class, dose and treatment duration in complete agreement with the guidelines
Guidelines not followed	Antimicrobial use/non-use not in agreement with the guidelines, i.e. different dose^c^ or treatment duration^d^, different antimicrobial class^e^ or no antibiotics prescribed despite being indicated
**AMU**^**b**^**not indicated**	
Guidelines followed	No antibiotics prescribed
Guidelines not followed	Unjustified prescription of antibiotics

^a^Based on the Justification Scores in Schmitt et al., 2019 [45]; ^b^AMU, antimicrobial use; ^c^A deviation of up to 20% above or below the recommended dose was accepted; ^d^A margin of one day shorter or longer was tolerated; ^e^Cases were only listed once, i.e. if dose or treatment duration as well as antimicrobial class deviated from the guidelines, cases were listed in “different antimicrobial class”

For statistical analysis, IBM SPSS Statistics 23® (IBM, New York, USA) and the software R version 3.6.1 (R Foundation for Statistical Computing, Vienna, Austria [68]) were used. A Mann-Whitney U Test was performed to compare continuous data such as age and treatment duration between the two evaluation periods. Categorical variables (university hospital versus private practice, sex, breed, pretreatment, hospitalization, diagnostic work-up, local wound treatment, drainage, indication for antimicrobial use, antimicrobial treatment, prescribed antimicrobial classes and use of HPCIAs, serial or combination therapy) were presented as proportions with Clopper Pearson 95% CI, which were calculated with the command binom.test() [68]. A mixed effects logistic regression model (lme4 package [69]) was used to compare compliance with the guidelines between 2016 and 2018 and between the disease complexes (aURTD, FLUTD, abscess). Analysis was done separately for cases with and without indication for antimicrobial use (Table 6). The binary outcome consisted of whether or not a prescription was in agreement with the guidelines. In the multivariable model, year and indication (aURTD, FLUTD or abscesses), were included as fixed effects. Based on our research question and expert knowledge, we decided to include both explanatory variables in the multivariable model. Additionally, an interaction between year and indication was tested with a likelihood ratio test with the package lmtest (cit) [70]. The different hospitals and practices were included as random effects.

## Supplementary information

**Additional file 1.** Age distribution in 2016 and 2018 for cats with aURTD, FLUTD and abscesses.

**Additional file 2.** Antimicrobial prescriptions in 2016 and 2018 and separated for university hospitals and private practices.

**Additional file 3.** Prescribed combination therapies in 2016 and 2018 in cats with aURTD, FLUTD and abscesses.

**Additional file 4.** Compliance with guidelines in 2016 and 2018 and separated for university hospitals and private practices.

## Data Availability

The datasets used and analyzed during the current study are available from the corresponding author on reasonable request.

## Abbreviations

aURTD: Acute upper respiratory tract disease
FLUTD: Feline lower urinary tract disease
CI: Confidence interval
OR: Odds ratio
HPCIA: Highest priority critically important antimicrobial
StAR: Strategy on Antimicrobial Resistance
vs.: Versus
PCR: Polymerase chain reaction
WHO: World Health Organization
AMU: Antimicrobial use
NA: Not applicable
SID: Once daily
BID: Twice daily
TID: Three times daily
UTI: Urinary tract infection
