# Supplementary material for: Antimicrobial prescriptions in cats in Switzerland before and after the introduction of an online antimicrobial stewardship tool
Source: BMC Vet Res. 2020 Jul 3;16:229. doi: 10.1186/s12917-020-02447-8 (PMC7333330; doi:10.1186/s12917-020-02447-8)
Supplement: Supplementary file 1 — Additional file 1. Age distribution in 2016 and 2018 for cats with aURTD, FLUTD and abscesses. [file 12917_2020_2447_MOESM1_ESM.pdf]

Additional file 1: Age distribution in 2016 and 2018 for cats with aURTD[a], FLUTD[b] and abscesses.

Parameter	aURTD[a]			FLUTD[b]			Abscesses		
	2016	2018		2016	2018		2016	2018	
**Numbers of cases**	n = 219	n = 234		n = 328	n = 315		n = 209	n = 217	
	Median (range)	Median (range)	*p*-value	Median (range)	Median (range)	*p*-value	Median (range)	Median (range)	*p*-value
Age[c] (years)	3 (0.04-19)	6 (0.08-21)	0.002	8 (0.17-21)	8 (0.25-22)	0.899	7 (0.50-18)	7 (0.03-20)	0.772

Data from cases from 2016 has been published previously (1); [a]aURTD, acute upper respiratory tract disease; [b]FLUTD, feline lower urinary tract disease; [c]Age is unknown for numbers not listed

References

1. Schmitt K, Lehner C, Schuller S, Schüpbach-Regula G, Mevissen M, Peter R, et al. Antimicrobial use for selected diseases in cats in Switzerland. BMC Vet Res. 2019;15(1):94.
