# Supplementary material for: Antimicrobial prescriptions in cats in Switzerland before and after the introduction of an online antimicrobial stewardship tool
Source: BMC Vet Res. 2020 Jul 3;16:229. doi: 10.1186/s12917-020-02447-8 (PMC7333330; doi:10.1186/s12917-020-02447-8)
Supplement: Supplementary file 2 — Additional file 2. Antimicrobial prescriptions in 2016 and 2018 and separated for university hospitals and private practices. [file 12917_2020_2447_MOESM2_ESM.pdf]

Additional file 2: Antimicrobial prescriptions in 2016 and 2018 and separated for university hospitals and private practices.

Parameter		Total		University hospitals		Private practices	
		2016	2018	2016	2018	2016	2018
**Total number of cases**		n = 776	n = 792	n = 173	n = 144	n = 603	n = 648
		% [CI][a]	% [CI][a]	% [CI][a]	% [CI][a]	% [CI][a]	% [CI][a]
Antimicrobial treatment	Yes[b]	**75.0 [71.8-78.0]**	**66.7 [63.3-69.9]**	**67.1 [59.5-74.0]**	**49.3 [40.9-57.8]**	77.3 [73.7-80.6]	70.5 [66.8-74.0]
**Details of antimicrobial treatment**		n = 582	n = 528	n = 116	n = 71	n = 466	n = 457
		% [CI][a]	% [CI][a]	% [CI][a]	% [CI][a]	% [CI][a]	% [CI][a]
Pot. aminopenicillin		55.5 [51.4-59.6]	60.8 [56.5-65.0]	85.3 [77.6-91.2]	84.5 [74.0-92.0]	48.1 [43.4-52.7]	57.1 [52.4-61.7]
3rd generation cephalosporin		26.1 [22.6-29.9]	19.5 [16.2-23.1]	7.8 [3.6-14.2]	2.8 [0.3-9.8]	**30.7 [26.5-35.1]**	**22.1 [18.4-26.2]**
Aminopenicillin		**19.6 [16.4-23.0]**	**27.8 [24.1-31.9]**	1.7 [0.2-6.1]	5.6 [1.6-13.8]	24.0 [20.2-28.2]	31.3 [27.1-35.8]
Fluoroquinolone		6.4 [4.5-8.7]	8.7 [6.4-11.4]	5.2 [1.9-10.9]	8.5 [3.2-17.5]	6.7 [4.6-9.3]	8.8 [6.3-11.7]
Tetracycline		5.0 [3.4-7.1]	6.6 [4.7-9.1]	2.6 [0.5-7.4]	4.2 [0.9-11.9]	5.6 [3.7-8.1]	7.0 [4.8-9.7]
1st generation cephalosporin		3.3 [2.0-5.1]	4.0 [2.5-6.0]	4.3 [1.4-9.8]	2.8 [0.3-9.8]	3.0 [1.7-5.0]	4.2 [2.5-6.4]
Others[c]		2.2 [1.2-3.8]	2.7 [1.5-4.4]	0.9 [0.0-4.7]	2.8 [0.3-9.8]	2.6 [1.3-4.5]	2.6 [1.4-4.5]
HPCIAs[d]	Yes[b]	33.0 [29.2-37.0]	28.8 [25.0-32.9]	12.9 [7.4-20.4]	12.7 [6.0-22.7]	38.0 [33.6-42.6]	31.3 [27.1-35.8]
Serial/combination therapy	Yes[b]	**17.0 [14.0-20.3]**	**27.7 [23.9-31.7]**	8.6 [4.2-15.3]	11.3 [5.0-21.0]	**19.1 [15.6-23.0]**	**30.2 [26.0-34.6]**

Non-overlapping 95% confidence intervals are shown in bold; Data from cases from 2016 has been published previously (1); [a]CI, 95% confidence interval; [b]Values for the category "no" (reference group) are not shown; [c]Others (antimicrobial classes used in ≤2% of prescriptions) included amphenicoles, lincosamides, macrolides, penicillins, nitroimidazoles; [d]HPCIAs, highest priority critically important antimicrobials

References

1. Schmitt K, Lehner C, Schuller S, Schüpbach-Regula G, Mevissen M, Peter R, et al. Antimicrobial use for selected diseases in cats in Switzerland. BMC Vet Res. 2019;15(1):94.
