# Supplementary material for: Antimicrobial prescriptions in cats in Switzerland before and after the introduction of an online antimicrobial stewardship tool
Source: BMC Vet Res. 2020 Jul 3;16:229. doi: 10.1186/s12917-020-02447-8 (PMC7333330; doi:10.1186/s12917-020-02447-8)
Supplement: Supplementary file 3 — Additional file 3. Prescribed combination therapies in 2016 and 2018 in cats with aURTD, FLUTD and abscesses. [file 12917_2020_2447_MOESM3_ESM.pdf]

Additional file 3: Prescribed combination therapies in 2016 and 2018 in cats with aURTD, FLUTD and abscesses.

	2016		2018	
**Indication**	**Combination of antibiotic classes**	**n=**	**Combination of antibiotic classes**	**n=**
**aURTD**[a]	Aminopenicillin + amphenicol	2	Aminopenicillin + tetracycline	3
	Aminopenicillin + tetracycline	1	Aminopenicillin + fluorochinolone	3
	Aminopenicillin + 1st generation cephalosporin	1	Aminopenicillin + 3rd generation cephalosporin	1
	Aminopenicillin + 3rd generation cephalosporin	1	Tetracycline + macrolide	1
	Aminopenicillin + fluoroquinolone	1	Fluoroquinolone + amphenicol	1
	Aminopenicillin + fluoroquinolone + tetracycline	1	Fluoroquinolone + tetracycline	1
	Potentiated aminopenicillin + fluoroquinolone	1		
	**Total number of combination therapies**	**8**	**Total number of combination therapies**	**10**
**FLUTD**[b]	Aminopenicillin + 3rd generation cephalosporin	1	Aminopenicillin + fluoroquinolone	3
	Aminopenicillin + fluoroquinolone	1	Potentiated aminopenicillin + fluoroquinolone	3
	Potentiated aminopenicillin + fluoroquinolone	1		
	**Total number of combination therapies**	**3**	**Total number of combination therapies**	**6**
**Abscesses**	Aminopenicillin + 3rd generation cephalosporin	2	Aminopenicillin + 3rd generation cephalosporin	4
	Potentiated aminopenicillin + fluoroquinolone	1	Potentiated aminopenicillin + fluoroquinolone	2
			Aminopenicillin + 3rd generation cephalosporin + fluoroquinolone	1
			Aminopenicillin + potentiated aminopenicillin	1
			Aminopenicillin + fluoroquinolone	1
			Potentiated aminopenicillin + 1st generation cephalosporin	1
			1st generation cephalosporin + fluoroquinolone	1
	**Total number of combination therapies**	**3**	**Total number of combination therapies**	**11**

Data from cases from 2016 has been published previously (1); [a]aURTD, acute upper respiratory tract disease; [b]FLUTD, feline lower urinary tract disease

References

1. Schmitt K, Lehner C, Schuller S, Schüpbach-Regula G, Mevissen M, Peter R, et al. Antimicrobial use for selected diseases in cats in Switzerland. BMC Vet Res. 2019;15(1):94.
