# Supplementary material for: Antimicrobial prescriptions in cats in Switzerland before and after the introduction of an online antimicrobial stewardship tool
Source: BMC Vet Res. 2020 Jul 3;16:229. doi: 10.1186/s12917-020-02447-8 (PMC7333330; doi:10.1186/s12917-020-02447-8)
Supplement: Supplementary file 4 — Additional file 4. Compliance with guidelines in 2016 and 2018 and separated for university hospitals and private practices. [file 12917_2020_2447_MOESM4_ESM.pdf]

Additional file 4: Compliance with guidelines in 2016 and 2018 and separated for university hospitals and private practices.

Category	Total		University hospital		Private practices	
	2016	2018	2016	2018	2016	2018
**AMU[a] indicated**	n = 186	n = 215	n = 71	n = 45	n = 115	n = 170
	% [CI][b]	% [CI][b]	% [CI][b]	% [CI][b]	% [CI][b]	% [CI][b]
Guidelines followed	33.3 [26.6-40.6]	33.5 [27.2-40.2]	26.8 [16.9-38.6]	42.2 [27.7-57.8]	37.4 [28.5-46.9]	31.2 [24.3-38.7]
Guidelines not followed						
a. Different dose/duration	11.3 [7.1-16.7]	15.3 [10.8-20.9]	2.8 [0.3-9.8]	8.9 [2.5-21.2]	16.5 [10.3-24.6]	17.1 [11.7-23.6]
b. Different antimicrobial class	50.0 [42.6-57.4]	42.8 [36.1-49.7]	**67.6 [55.5-78.2]**	**33.3 [20.0-49.0]**	39.1 [30.2-48.7]	45.3 [37.7-53.1]
c. Unjustified non-use	5.4 [2.6-9.7]	8.4 [5.0-12.9]	2.8 [0.3-9.8]	15.6 [6.5-29.5]	7.0 [3.1-13.2]	6.5 [3.3-11.3]
**AMU[a] not indicated**	n = 292	n = 248	n = 90	n = 74	n = 202	n = 174
	% [CI][b]	% [CI][b]	% [CI][b]	% [CI][b]	% [CI][b]	% [CI][b]
Guidelines followed	**35.6 [30.1-41.4]**	**54.0 [47.6-60.4]**	55.6 [44.7-66.0]	73.0 [61.4-82.6]	**26.7 [20.8-33.4]**	**46.0 [38.4-53.7]**
**Treatment duration[c]**	n = 471	n = 425	n = 109	n = 65	n = 362	n = 360
	% [CI][b]	% [CI][b]	% [CI][b]	% [CI][b]	% [CI][b]	% [CI][b]
Guidelines followed	56.1 [51.4-60.6]	58.8 [54.0-63.5]	61.5 [51.7-70.6]	67.7 [54.9-78.8]	54.4 [49.1-59.6]	57.2 [51.9-62.4]
Guidelines not followed						
a. Too long	41.0 [36.5-45.6]	36.9 [32.3-41.7]	36.7 [27.7-46.5]	18.5 [9.9-30.0]	42.3 [37.1-47.5]	40.3 [35.2-45.5]
b. Too short	3.0 [1.6-4.9]	4.2 [2.5-6.6]	**1.8 [0.2-6.5]**	**13.8 [6.5-24.7]**	3.3 [1.7-5.7]	2.5 [1.1-4.7]

Non-overlapping 95% confidence intervals are shown in bold; Data from cases from 2016 has been published previously (1); [a]AMU, antimicrobial use; [b]CI, 95% confidence intervall; [c]Treatment duration is unknown or not applicable for numbers not listed

References

1. Schmitt K, Lehner C, Schuller S, Schüpbach-Regula G, Mevissen M, Peter R, et al. Antimicrobial use for selected diseases in cats in Switzerland. BMC Vet Res. 2019;15(1):94.
